# GmMYB114 Facilitates the Synthesis of Anthocyanins in Soybean Sprouts under Blue Light

**DOI:** 10.3390/plants13081107

**Published:** 2024-04-16

**Authors:** Li Jia, Hong Xu, Xinxin Xu, Kai Gao, Keying Zhao, Jingran Dong, Nana Su

**Affiliations:** 1 College of Food and Drug, Luoyang Normal University, Luoyang 471934, China; jialilynu@sina.com; 2 College of Life Science, Nanjing Agriculture University, Nanjing 210095, China; 9211010221@stu.njau.edu.cn (X.X.); 9211010222@stu.njau.edu.cn (K.G.); 9211010219@stu.njau.edu.cn (K.Z.); 9211010224@stu.njau.edu.cn (J.D.); 3 College of Science, Minzu University of China, Beijing 100086, China; hongxulalala@163.com

**Keywords:** soybean sprouts, blue light, anthocyanins, GmMYB114

## Abstract

Soybean sprouts constitute a significant segment of the vegetable market due to their nutritional richness, particularly in various flavonoids, which contribute to numerous health benefits. The augmentation of the flavonoid content in soybean sprouts is pivotal for enhancing their economic value. While research has established the potential of blue light in promoting the synthesis of anthocyanins, a subclass of flavonoids known for their health advantages, the precise regulatory mechanisms remain elusive. In this study, we identified a notable upregulation of an R2R3 type MYB transcription factor, GmMYB114, in response to blue light exposure, exhibiting a significant positive correlation with anthocyanin accumulation in soybean sprouts. The functional role of GmMYB114 was validated in soybean hairy roots, wherein its overexpression substantially augmented anthocyanin synthesis. Further investigations employing yeast one-hybrid (Y1H), dual-luciferase reporter (LUC), and GUS assays revealed that GmMYB114 indirectly influences anthocyanin synthesis as it does not directly bind to the promoters of anthocyanin synthesis genes to activate their expression. These findings contribute to elucidating the mechanism underlying blue light-mediated enhancement of anthocyanin synthesis in soybean sprouts, offering valuable insights for harnessing molecular technologies to obtain anthocyanin-enriched soybean sprouts.

## 1. Introduction

Sprouts are kinds of important components that cannot be ignored in the nowadays vegetable market. Studies have shown that after seed germination, the nutritional components, including the protein content, vitamin content, and flavonoid compound content, will significantly increase [1,2,3]. The simple acquisition method combined with unique taste has led to an increasing number of species being developed into sprout vegetables. Among them, soybeans, usually an important oil-seed crop, occupy a significant position in the vegetable market in the form of sprouts, especially in East Asian countries [4].

Soybean sprouts contain abundant nutrients and bioactive substances, such as protein [5], vitamin C [6], trace elements [7], and flavonoids [8], which endow soybean sprouts with important functions such as antioxidant [9]), antibacterial [10], anticancer [11], and anti-inflammatory functions [12]. Soybean sprouts contain abundant flavonoids, including anthocyanins, flavonols, and isoflavones [13,14]. As the main type of flavonoids, anthocyanins have strong antioxidant capacity [15]. Anthocyanins, composed of anthocyanidins and carbohydrates, are a widely present flavonoid compound in plants [16]. As a kind of natural pigment substance, anthocyanins can impart various colors to the stems, leaves, flowers, and fruits of plants, with red, blue, and purple being the main colors [17]. It is reported that there are over 250 types of anthocyanins present in nature. Among them, more than 20 types of anthocyanins have been identified, but only 6 are the most common: cyanidin, pelargonidin, delphinnidin, peonidin, petunidin, and malvidin [18].

With the increasing attention on anthocyanins, the biosynthesis pathway of anthocyanins has been studied more clearly. Overall, anthocyanins are synthesized in the cytoplasm through the flavonoid branch of the phenylpropanoid metabolism pathway and then transported to vacuoles for storage [19]. The key synthetase genes in the synthesis process mainly include (in upstream and downstream order) the *phenylalanine ammonia lyase gene* (*PAL*), *cinamate 4-hydroxylase gene* (C4H), *4-coalate CoA ligase gene* (*4CL*), *chalcone synthesis gene* (*CHS*), *chalcone isomerase gene* (*CHI*), *flavonone 3-hydroxylase gene* (*F3H*), *flavonoid 3′-hydroxylase gene* (*F3′H*), *dihydroflavonol 4-reductase gene* (*DFR*), *anthocyanidin synthesis gene* (*ANS*), and *flavonoid-3-O-glucosyltransfer gene* (*UFGT*). The regulation of anthocyanin synthesis is generally based on the regulation of these key synthetic genes.

The biosynthesis of anthocyanins is regulated by a series of transcription factors, which mainly regulate anthocyanin synthesis in two ways: (1) by directly binding to cis-acting elements in the promoter of anthocyanin biosynthesis genes, thereby regulating gene expression at the transcriptional level; (2) The formation of protein complexes between transcription factors synergistically regulates the synthesis of anthocyanins. The MBW protein ternary complex composed of the MYB transcription factor, the bHLH transcription factor, and the WD40 transcription factor is the most classic way to regulate anthocyanin synthesis in plants [20]. Among them, MYB and bHLH can not only participate in the formation of complexes but also act independently.

The synthesis of anthocyanins in plants is not only regulated by a series of transcription factors but also by other factors, mainly environmental and hormonal factors [21,22]. Among the environmental factors, light is one of the biggest factors affecting the accumulation of anthocyanins, and most plants require the participation of light to synthesize anthocyanins [23]. Blue light has been reported to induce the synthesis of anthocyanins in soybean sprouts, but its mechanism still needs further investigation [24,25]. Therefore, this paper identified a potential MYB transcription factor, GmMYB114, that responds to blue light regulation of soybean sprout anthocyanin synthesis by screening the blue light transcriptome and preliminarily exploring its regulation mechanism. Our research can provide a theoretical basis for revealing the regulation of anthocyanin synthesis in soybean sprouts by light and provide an experimental basis for improving the nutritional and economic value of soybean sprouts.

## 2. Results

### 2.1. Blue Light Promotes the Synthesis of Anthocyanins in Soybean Sprouts

**Figure 1:**
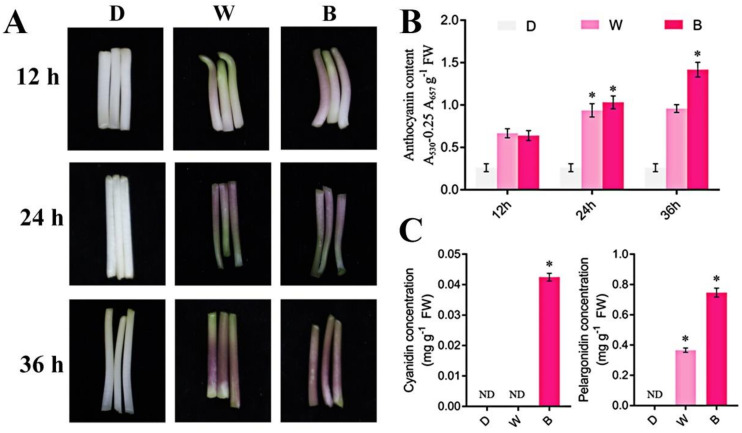
**Blue light induces anthocyanin synthesis in soybean sprouts.** (**A**) Anthocyanin synthesis phenotype of soybean sprouts after dark (D)/white light (W)/blue light (B) treatment for 12 h/24 h/36 h. (**B**) Determination of anthocyanin content in soybean sprouts after dark (D)/white light (W)/blue light (B) treatment for 12 h/24 h/36 h. (**C**) Determination of anthocyanin monomers, including cyanidin and pelargonidin concentration, in soybean sprouts after dark (D)/white light (W)/blue light (B) treatment for 36 h using liquid chromatography-mass spectrometry. * *p* < 0.05. ND indicates not detected.

We verified the positive regulatory effect of blue light on the synthesis of anthocyanins in soybean sprouts. Firstly, compared to darkness, white light treatment can promote the synthesis of anthocyanins in soybean sprouts (Figure 1A). Considering that white light is mainly composed of blue and red light, we also treated soybean sprouts with different light qualities of the same light intensity. It was found that blue light can significantly increase the content of anthocyanins in soybean sprouts, while red light cannot (Figure 1A and Figure S1). The determination results of anthocyanins in soybean sprouts under different light qualities confirmed this observation in Figure 1A (Figure 1B,C).

### 2.2. Identification of GmMYB114 as a Positive Regulator of Anthocyanins in Soybean Sprouts Response to Blue Light

**Figure 2:**
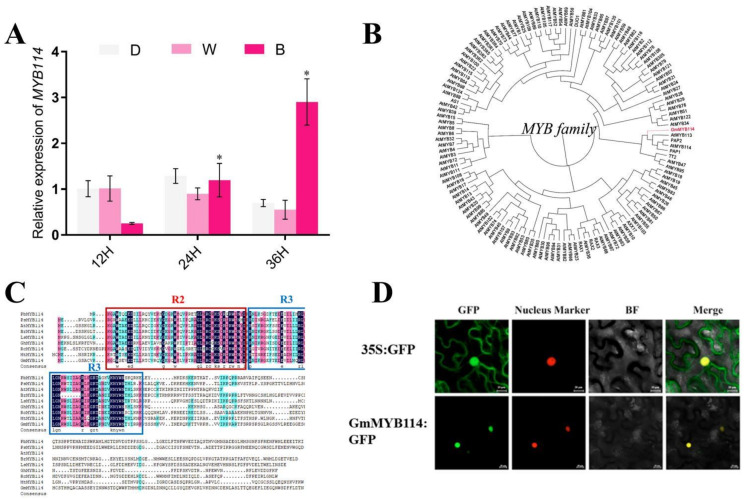
**Identification of GmMYB114.** (**A**) Expression level of *GmMYB114* in soybean sprouts after dark/white light/blue light treatment for 12 h/24 h/36 h. (**B**) Phylogenetic tree analysis of GmMYB114 with all MYBs in *Arabidopsis thaliana*. (**C**) Amino acid sequence analysis of GmMYB114 (*Glycine max*, XP_003549924) and MYB114s in other plant species including LsMYB114 (*Lathyrus sativus*, CAK8562714), PsMYB114 (*Pisum sativum*, XP_050871457.1), MtMYB114 (*Medicago truncatula*, XP_039688941), GhMYB114 (*Gossypium hirsutum*, XP_040952376.1), AtMYB114 (*Arabidopsis thaliana*, AEE34502.1), BrMYB114 (*Brassica rapa*, XP_009135860.2), PbMYB114 (*Pyrus x bretschneideri*, XP_048443068.1), and RcMYB114 (*Rosa chinensis*, XP_024185366.1). (**D**) Subcellular localization assay of GmMYB114. D, darkness; W, white light; B, blue light. * *p* < 0.05. BF, bright field.

To investigate the mechanism of blue light regulation of anthocyanin synthesis in soybean sprouts, we analyzed the transcriptome of soybean sprouts under blue light [24]. We identified a differentially expressed MYB transcription factor, GmMYB114, from the transcriptome (Figure S2). The MYB family transcription factors account for the transcription factor class containing the largest number of differentially expressed genes in the transcriptome, with *GmMYB114* showing the most significant changes in expression. Therefore, we chose this gene for further research. The expression level of *GmMYB114* was validated using RT-qPCR, and it was found that its expression level significantly increased under blue light (Figure 2A). Its expression pattern was positively correlated with anthocyanin synthesis (Figure S3). An evolutionary tree was constructed using all the MYBs in *Arabidopsis thaliana* (Figure 2B), and it was found that GmMYB114 clustered together with AtMYB113, AtMYB114, PAP1, and PAP2, which have been reported to participate in regulating anthocyanin synthesis in *Arabidopsis thaliana*. The results of amino acid sequence analysis also showed that GmMYB114 has a conserved R2 and R3 domain of R2R3-MYB family transcription factors (Figure 2C). We also performed a subcellular localization assay on GmMYB114 and found that it is located in the nucleus (Figure 2D).

### 2.3. GmMYB114 Can Promote the Synthesis of Anthocyanins in Soybean Hairy Roots

**Figure 3:**
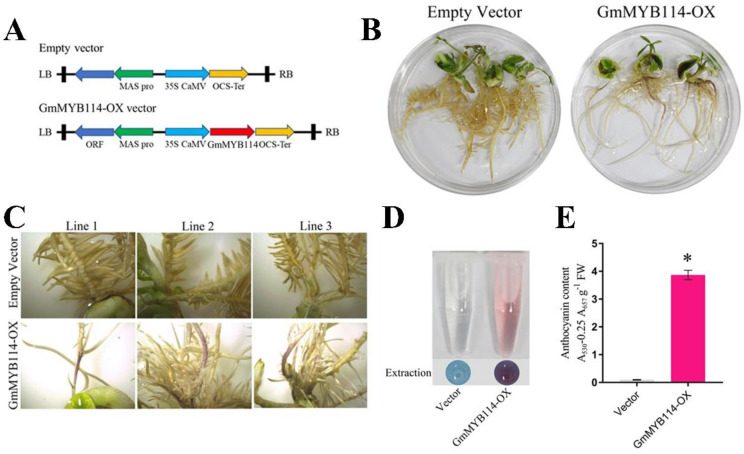
**GmMYB114 promotes anthocyanin synthesis in soybean hairy roots.** (**A**) Schematic diagram of vector construction in soybean hairy root transformation. (**B**) Soybean hairy root phenotypes transformed with empty vector (EV) and overexpressed GmMYB114 (GmMYB114−OX). (**C**) Partial enlargement of anthocyanins in three independent soybean hairy root lines transformed with empty vector (EV) and overexpressed GmMYB114 (GmMYB114−OX). (**D**) Anthocyanin extracts from soybean hairy roots transformed with empty vector (EV) and overexpressed GmMYB114 (GmMYB114−OX). (**E**) Determination of anthocyanin content in soybean hairy roots transformed with empty vector (EV) and overexpressed GmMYB114 (GmMYB114−OX). * *p* < 0.05.

After screening and identifying GmMYB114, we validated its ability to promote anthocyanin synthesis in vivo using the soybean hairy root system. We constructed soybean hairy roots that were transformed with empty vector and soybean hairy roots that were overexpressed with *GmMYB114*, respectively (Figure 3A) and demonstrated the success of the transformation through different PCR methods (Figure S4). The results showed that compared with the hairy roots transformed with the empty vector, the surface of the hairy roots overexpressing *GmMYB114* showed deposition of red pigments (Figure 3B,C). After determining the anthocyanin content of all the materials, it was found that the overexpression of *GmMYB114* significantly increased the anthocyanin content in soybean hairy roots (Figure 3D).

### 2.4. Overexpression of GmMYB114 Can Promote the Expression of Anthocyanin Synthesis Genes

**Figure 4:**
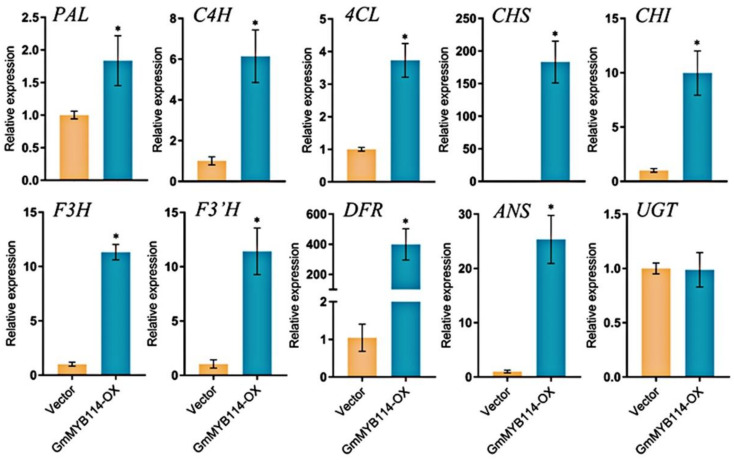
**Overexpression of GmMYB114 increased the expression of anthocyanin synthesis genes in hairy roots.** Determination of gene expression in the anthocyanin synthesis pathway in soybean hairy roots transformed with (EV) and overexpressed GmMYB114 (GmMYB114−OX), including *GmPAL*, *GmC4H*, *Gm4CL*, *GmCHS*, *GmCHI*, *GmF3H*, *GmF3′H*, *GmDFR*, *GmANS*, and *GmUGT*. * *p* < 0.05.

After successfully constructing hairy roots overexpressing *GmMYB114*, we also detected the expression of genes related to anthocyanin synthesis in different materials. The RT-qPCR results showed that after the overexpression of *GmMYB114*, the expression levels of most genes in the anthocyanin synthesis pathway were significantly increased, including *GmPAL*, *GmC4H*, *Gm4CL*, *GmCHS*, *GmCHI*, *GmF3H*, *GmF3′H*, *GmDFR*, and *GmANS* (Figure 4). Among them, the expression of *GmCHS*, *GmDFR*, and *GmANS* showed the most significant increase, so we speculated that GmMYB114 may increase the synthesis of anthocyanins in soybean sprouts by promoting the expression of these genes.

### 2.5. GmMYB114 Cannot Directly Regulate the Expression of GmCHS, GmDFR, and GmANS

**Figure 5:**
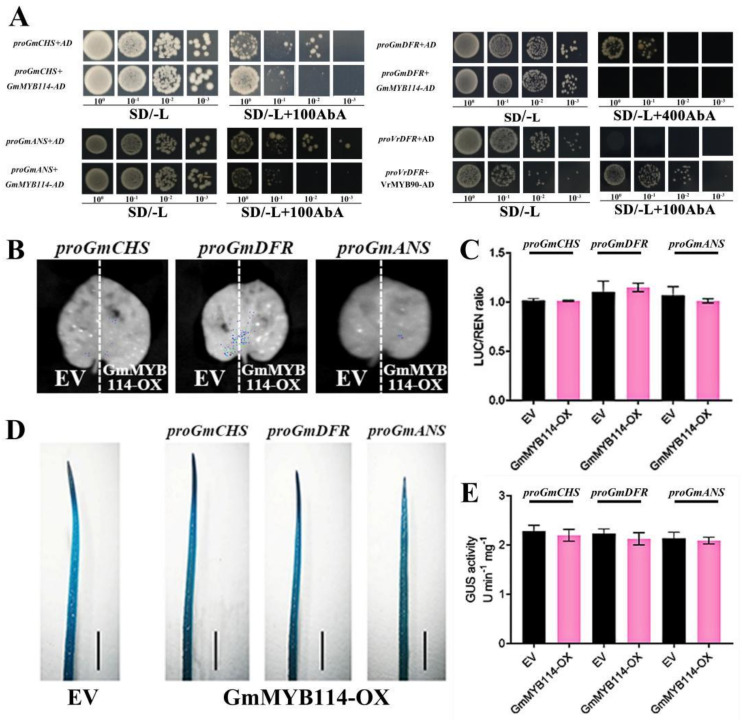
**GmMYB114 cannot directly promote the expression of anthocyanin synthesis genes.** (**A**) Yeast 1-hybrid (Y1H) experiment showed that GmMYB114 could not bind to the promoters of *GmCHS*, *GmDFR*, or *GmANS*. Different numbers with a base of 10 under the yeast pictures represent the multiples of yeast dilution. AbA, aureobasidin A. (**B**) LUC analysis of GmMYB114 on the expression of *GmCHS*, *GmDFR*, and *GmANS*. The left side of the tobacco leaf was injected with *Agrobacterium* transformed with empty vector (EV), while the right side was injected with *Agrobacterium* transformed with GmMYB114 (GmMYB114−OX). (**C**) Determination of LUC/REN ratio in (**B**). (**D**) GUS analysis of GmMYB114 on the expression of *GmCHS*, *GmDFR*, and *GmANS* using soybean hairy roots. Hairy roots transformed with empty vector (EV) and GmMYB114 (GmMYB114−OX) were used for GUS staining. (**D**) shows the final result of GUS staining, with darker blue indicating stronger activation effect. Bar = 0.1 cm (**E**) Determination of GUS activity in (**D**).

Considering that *GmCHS*, *GmDFR*, and *GmANS* may be regulated by GmMYB114, we conducted relevant validation experiments. Firstly, we analyzed the promoter sequences of these three genes and found potential MYB binding sites in all three promoter regions, including MRE (MYB recognition element) and G-box (Figure S5). We first used yeast heterozygosity technology to investigate whether GmMYB114 can bind to the promoter regions of these three genes. We used the VrMYB90-*proVrDFR* module, which was previously reported to generate interactions in the same Y1H system, as a positive control [26]. It was found that under the conditions of this experiment, VrMYB90 could bind to the promoter of *VrDFR*. The results showed that although there were MYB binding sites in the promoter region, GmMYB114 could not bind to the promoter of *GmCHS*/*GmDFR*/*GmANS* in the yeast system (Figure 5A). Next, we used LUC and GUS to investigate whether GmMYB114 promotes the expression of these three genes. The LUC results showed that compared with the control, GmMYB114 did not increase the expression of *GmCHS*/*GmDFR*/*GmANS* (Figure 5B,C). The results of GUS are also consistent with those of LUC (Figure 5D,E).

## 3. Discussion

Soybean sprouts are rich in flavonoids, which are beneficial to human health as antioxidants. Further increasing the content of flavonoids in soybean sprouts can enhance their nutritional and even economic value. Our research findings, as well as those of others, indicated that blue light can promote an increase in the content of anthocyanins in soybean sprouts, one of the most important flavonoids [25,27,28]. We attempted to explore the potential mechanisms underlying this phenomenon and identified an R2R3-MYB transcription factor, GmMYB114, that can respond to blue light and regulate the synthesis of anthocyanins in soybean sprouts. The expression level of *GmMYB114* was positively correlated with the changes in anthocyanin content in soybean sprouts under blue light. It has also been confirmed in soybean hairy roots that GmMYB114 can promote anthocyanin synthesis by increasing the expression of key genes in the anthocyanin synthesis pathway. Furthermore, we found that GmMYB114 cannot directly regulate the expression of anthocyanin synthesis genes *GmCHS*/*GmDFR*/*GmANS* but rather regulates the synthesis of anthocyanins in soybean sprouts induced by blue light.

The MYB family of transcription factors has been reported to be one of the most classic transcription factor families that regulate plant anthocyanin synthesis. The transcription factors AtMYB75/PAP1 (production of anthocyanin segment 1) [29], AtMYB90/PAP2 (production of anthocyanin segment 2) [30], AtMYB113 [31], and AtMYB114 [32] in SG6 of *Arabidopsis* regulate *AtDFR* and *AtANS*, thereby affecting the synthesis of anthocyanins. In recent years, research has been conducted in-depth on MYB transcription factors involved in anthocyanin biosynthesis in *Arabidopsis*, and many MYBs regulating anthocyanin synthesis in horticultural plants and crops have also been reported, such as VvMYB5a [33], VvMYB5b [34], and VvMYBAl [35] in *Vitis vinifera*; MdMYB10 [36], MdMYBAl, and MdMYBA [37] in *Malus domestica*; PyMYB10 and PyMYB114 in *Pyrus pyrifolia* [38,39]; and RsMYB1 in *Raphanus sativus* [40]. In soybean (*Glycine max*), some MYB transcription factors have also been reported to regulate the synthesis of anthocyanins, such as GmMYBA2 and GmR [41]. Here, we identified a MYB transcription factor, GmMYB114, in soybean that has not yet been reported to function. It is closely related to AtMYB114 in *Arabidopsis* and has a typical conserved domain of R2R3. In our study, we also confirmed that GmMYB114 has the function of regulating anthocyanins.

However, in further research, we showed that GmMYB114 can regulate the expression of genes involved in the anthocyanin synthesis pathway. Regulating the expression of genes involved in the anthocyanin synthesis pathway is the core of regulating anthocyanin synthesis in plants, including MYB transcription factors. In addition to this direct regulation of anthocyanin synthesis, there are other indirect ways to regulate plant anthocyanins. In *Arabidopsis*, PAP1/PAP2 can form MBW complexes with bHLH family TT8 and WD40 family TTG1 to jointly regulate the synthesis of anthocyanins [42]. In corn, the bHLH protein relies on MYB family C1 and WD40 family PAC1, thereby regulating the anthocyanin synthesis pathway [43] In eggplant, there are complex interactions between SmTTG1, SmGL3, and SmTT8, forming an MBW complex to jointly regulate the synthesis of anthocyanins [44]. Therefore, we speculated that GmMYB114 regulates anthocyanin synthesis by enhancing the function of MBW complexes with bHLH and WD40 family transcription factors. This regulatory approach has an indirect effect.

## 4. Materials and Methods

### 4.1. Plant Materials and Growth Conditions

The variety ‘DN690’ was used as the experimental material in this study. Complete soybean seeds were selected and soaked in deionized water at room temperature for 6–7 h. Then, all the seeds were placed in a seedling pot and incubated in a dark incubator for 24 h (25 °C ± 1 °C) to induce germination. After seed germination, soybean sprouts with consistent growth and uniform size were selected in a 96-well hydroponic box (with deionized water as the culture medium). The parameter settings of the light incubator in different light quality treatment experiments were as follows: the light quality was dark, blue, and red light, respectively; the light intensity was 25 μmol·m^−2^·s^−1^; the temperature was 25 ± 1 °C.

*Nicotiana benthamiana* was also used in the study. Tobacco seeds were sprinkled on suitable nutrient soil and grown under normal light cycles (16 h of light/8 h of darkness) at a culture temperature of 25 ± 1 °C and light intensity of 100 μmol·m^−2^·s^−1^ for 4–5 weeks. 

### 4.2. Extraction and Content Determination of Anthocyanins

Fresh 0.1 g samples of soybean hypocotyls treated with different light qualities were collected and cut into small sections. The sections were soaked in a 2 mL 1% HCl–methanol solution (*v*/*v*) and kept overnight at 25 °C in the dark. The OD value of the anthocyanins in the supernatant was measured using a UV spectrophotometer at the wavelengths 530 nm and 657 nm. The anthocyanin content was then calculated according to the following formula: anthocyanin content = (A530 − 0.25 × A657)/FW, where FW is the fresh weight (g).

### 4.3. Determination of Anthocyanin Monomers

The anthocyanin monomers were determined according to the method reported by Su et al. (2022) by liquid chromatography-mass spectrometry (Xevo G2-XS QTof, Waters, Milford, MA, USA). Briefly, 2 µL supernatant was injected to be tested with corresponding conditions, as follows: ACQUITY UPLCTMBEH C18 column (2.1 × 100 mm, 1.7 µm); the flow rate was 0.4 mL min^−1^; the mobile phase solvent A was 0.1% formic acid aqueous solution, and solvent B was 0.1% formic acid acetonitrile solution; the elution gradient was 0–2 min, 5% B/2–17 min, 5–95% B/17–19 min, 95% B/19–24 min, 95–5% B. Using an electrospray ionization (ESI) mass spectrometry ion source with a scanning range of 100–1200 m/z, a full scan was performed in positive ion mode. The ionization parameters included a capillary voltage of 2.0 kV, a collision voltage of 20–30 eV, an ion source temperature of 120 °C, and a drying temperature of 400 °C.

Cyanidin-3-O-glucoside (Yuanye, Shanghai, China) was used as the anthocyanin standard. Cyanin-3-O-glucoside was dissolved in methanol and gradient diluted to five concentrations of working solution: 0.0001/0.001/0.01/0.1/0.5 mg/mL. Then, the standard curve was established based on these five concentrations of working solutions using the method mentioned before. The content of the other monomers was calculated by comparing the concentrations of cyanidin-3-O-glucoside.

### 4.4. RNA Extraction and Real-Time Quantitative Real-Time Polymerase Chain Reaction (RT-qPCR) Analysis

A total of 1 g of soybean sprout hypocotyls treated with different light qualities stored at −80 °C was taken and ground into powder using liquid nitrogen and the total RNA was extracted using the Plant RNA Kit E.Z-N.A^®^ (Omega, Norcross, GA, USA). The integrity, concentration, and purity of the extracted RNA were verified using agarose gel electrophoresis, or the A260/230 value of the extracted RNA was measured. If the value was between 1.9 and 2.1, the purity of the RNA was good, and it was reserved for the next experiment. Then TOROBlue^®^ Qrt Premix with a gDNAEraser2.0 Kit (Toroivd, Shanghai, China) was used to reverse the RNA into cDNA.

Using 2xChamQTM Universal SYBR^®^, the qPCR Master Mix Kit (Vazyme, Nanjing, China) was used to detect the gene expression levels. Using *GmActin* as an internal reference gene and the 2^−ΔΔCT^ method, the relative expression level of the genes was calculated. All the primers used are shown in Supplemental Table S1.

### 4.5. Phylogenetic Tree and Amino Acid Sequence Analysis 

The protein sequence of GmMYB114 was compared with that of the MYBs in *Arabidopsis thaliana*, and *Arabidopsis* MYB genes were obtained from TAIR (shown in Supplemental Table S2). All the *MYB* genes were translated into protein sequences and aligned using Clustal X. Phylogenetic analysis was performed using MEGA X with neighbor-joining (NJ) criteria and verified using the maximum likelihood (ML) method. The 1000 bootstrap replicates were performed based on the multiple alignments of the protein sequences encoded by the *MYB* genes. NJ analysis was performed using the protein Poisson distances and the pairwise deletion of gap sites. The default parameters were used for ML analysis.

As for amino acid sequence analysis, the protein sequences of GmMYB114 (*Glycine max*, XP_003549924), LsMYB114 (*Lathyrus sativus*, CAK8562714), PsMYB114 (*Pisum sativum*, XP_050871457.1), MtMYB114 (*Medicago truncatula*, XP_039688941), GhMYB114 (*Gossypium hirsutum*, XP_040952376.1), AtMYB114 (*Arabidopsis thaliana*, AEE34502.1), BrMYB114 (*Brassica rapa*, XP_009135860.2), PbMYB114 (*Pyrus* × *bretschneideri*, XP_048443068.1), and RcMYB114 (*Rosa chinensis*, XP_024185366.1) were obtained from NCBI. The specific protein sequences can be found in Supplemental Table S3. Homologous alignment of all protein sequences was performed using DNAMAN.

### 4.6. Subcellular Localization Assay

Plasmids of pCAMBIA1305.1-GFP, pCAMBIA1305.1-GmMYB114-GFP, and a nuclear marker (MADS3-RFP) were, respectively, transferred into *Agrobacterium* strain GV3101 (Qingke, Beijing, China). The transient expression of vectors in *Nicotiana benthamiana* seedlings was performed according to the previously mentioned protocol [45]. Three days after infiltration, the undamaged epidermis of the leaves injected with a bacterial solution was selected for observation. The fluorescence was observed by laser confocal microscope at an excitation light of 488 nm for GFP and 546 nm for RFP (Zeiss LSM 710, Zeiss, Oberkochen, Germany), and then the images were photographed and saved.

### 4.7. Yeast One-Hybrid (Y1H) Assays

Y1H assays were performed using the Matchmaker Gold Yeast One-Hybrid System Kit according to the manufacturer’s protocols (Clontech, Shiga, Japan). The empty pGADT7 (AD) and GmMYB114-AD recombinant vector were constructed, and then these plasmids were linearized by restriction endonuclease BstBI (Biolabs, Ipswich, MA, USA) with 2 μL transfection of denatured salmon sperm DNA into 100 μL competent cells. The well-growing yeast was then selected to cultivate overnight at 30 °C. After that, the yeast with culture medium was centrifuged at 11,000 rpm for 1 min, washed twice with water, and the OD value was adjusted to 0.5–0.6. And, after gradient dilution, the resuspended yeast solution was dropped on SD/-Leu plates with aureobasidin A (AbA) of different concentrations (0, 100, 200, 400, and 600 mM) for 3–5 days of cultivation. If the yeast colony transferred with the GmMYB114-AD recombinant vector could grow normally on the culture medium with AbA added, this indicated that the GmMYB114 transcription factor could bind to the promoter of the structural gene. Conversely, if not, it indicated no interaction.

### 4.8. Dual Luciferase (LUC) Assays

The CDS sequence of GmMYB114 was constructed into the pGreenII62-SK vector, and the structural gene promoters were constructed into the pGreenII-0800-Luc vector. Then, the corresponding vectors were transformed into *Agrobacterium* and cultivated until the OD value was between 0.5 and 0.6. 

Tobacco plants that had grown for 4–5 weeks were selected, and the leaves were then injected with the *Agrobacterium* solution using a 1 mL syringe. The tobacco plants were cultivated overnight at 30 °C in darkness before being transferred to normal light conditions (16 h of light/8 h of darkness). Finally, the fluorescence was observed after 36 h. Then, the LUC signal value and REN signal value were calculated.

### 4.9. β-Glucuronidase Activity (GUS) Assays

For GUS analysis, following the procedure outlined by Zhan [45], the promoters were incorporated into the PCXGUS vector and then transferred into *Agrobacterium rhizogenes* K599. The *A. rhizogenes* strain K599 carrying both the effector and reporter was intercombined and transformed into soybean hairy roots.

### 4.10. Transient Expression Assays in Soybean Hairy Roots

For the transient transformation of soybean hairy roots [46], soybean seeds underwent sterilization using vacuum chlorine gas for 3–4 h. After germination on GM medium, the cut soybean cotyledon nodes were subjected to infection with *A. rhizogenes* strain K599 transformed with the constructed vector plasmid pFG5941-GmMYB114 for a duration of 30 min. Afterward, the explants were transferred to white solid medium for 5 d in the dark and for about 4 weeks in the light/dark condition. The method for extracting anthocyanins from hairy roots and determining their content was the same as the method for determining anthocyanins in plants, as described in Section 4.2.

### 4.11. Data Analysis

In this study, Excel 2016 was used to organize the data. SPSS 19.0 was used to analyze and process the data. GraphPad Prism was used to analyze the data and then acquire all the figures. All the experiments were conducted using more than three biological replicates.

## 5. Conclusions

Our study indicates that blue light can promote an increase in the content of anthocyanins in soybean sprouts. An R2R3-MYB transcription factor, GmMYB114, that can respond to blue light and regulate the synthesis of anthocyanins in soybean sprouts was identified in this study. Using soybean hairy roots, we demonstrated that GmMYB114 can promote anthocyanin synthesis by increasing the expression of key genes in the anthocyanin synthesis pathway. Furthermore, we found that GmMYB114 cannot directly regulate the expression of the anthocyanin synthesis genes *GmCHS*/*GmDFR*/*GmANS* but rather regulates the synthesis of anthocyanins in soybean sprouts induced by blue light in an indirect manner.

## Data Availability

Data are contained within the article.

## Supplementary Materials

The following supporting information can be downloaded at: https://www.mdpi.com/article/10.3390/plants13081107/s1, Figure S1: The effect of darkness (Dk)/blue light (BL)/red light (RL) on the synthesis of anthocyanins in soybean sprouts; Figure S2: Expression level of *GmMYB114* in transcriptome described in our previous study; Figure S3: The correlation between *GmMYB114* expression level and anthocyanin content; Figure S4: Verification of successful GmMYB114 transformation in soybean hairy roots; Figure S5: Analysis of MYB binding sites in the *GmCHS/GmDFR/GmANS* promoter region; Table S1: All primers used in this study; Table S2: All proteins used in the phylogenetic tree analysis; Table S3: All proteins used in the amino acid sequence analysis.
