# Supplementary material for: GmMYB114 Facilitates the Synthesis of Anthocyanins in Soybean Sprouts under Blue Light
Source: Plants (Basel). 2024 Apr 16;13(8):1107. doi: 10.3390/plants13081107 (PMC11055127; doi:10.3390/plants13081107)
Supplement: Supplementary file 1 [file plants-13-01107-s001.zip › Supporting information.pdf]

Supplemental figures

Figure S1 The effect of darkness (Dk)/blue light (BL)/red light (RL) on the synthesis of anthocyanins in soybean sprouts. $*p<0.05$.

Figure S2 Expression level of *GmMYB114* in transcriptome described in our previous study. The transcriptome was conducted between the soybean hypocotyl samples under dark (Dk) and blue light (BL) conditions. $*p<0.05$.

Figure S3 The correlation between *GmMYB114* expression level and anthocyanin content. The expression level of GmMYB114 was detected after being treated with blue light for 12 h, 24 h and 36 h. Also, the anthocyanin content was detected in the same time period. P indicated Pearson index, and the closer it is to 1, the more positive the correlation is.

Figure S4 Verification of successful *GmMYB114* transformation in soybean hairy roots. (A) The hairy roots transformed with empty vector and GmMYB114-OX vectoer were verified using primers with vector homologous arms to clone GmMYB114. The appearance of PCR bands indicated successful transfer to GmMYB114. (B) The expression level of *GmMYB114* in soybean hairy roots successfully detected in (A) was detected by RT qPCR. $*p<0.05$.

Figure S5 Analysis of MYB binding sites in the *GmCHS*/*GmDFR*/*GmANS* promoter region.

Figure S1

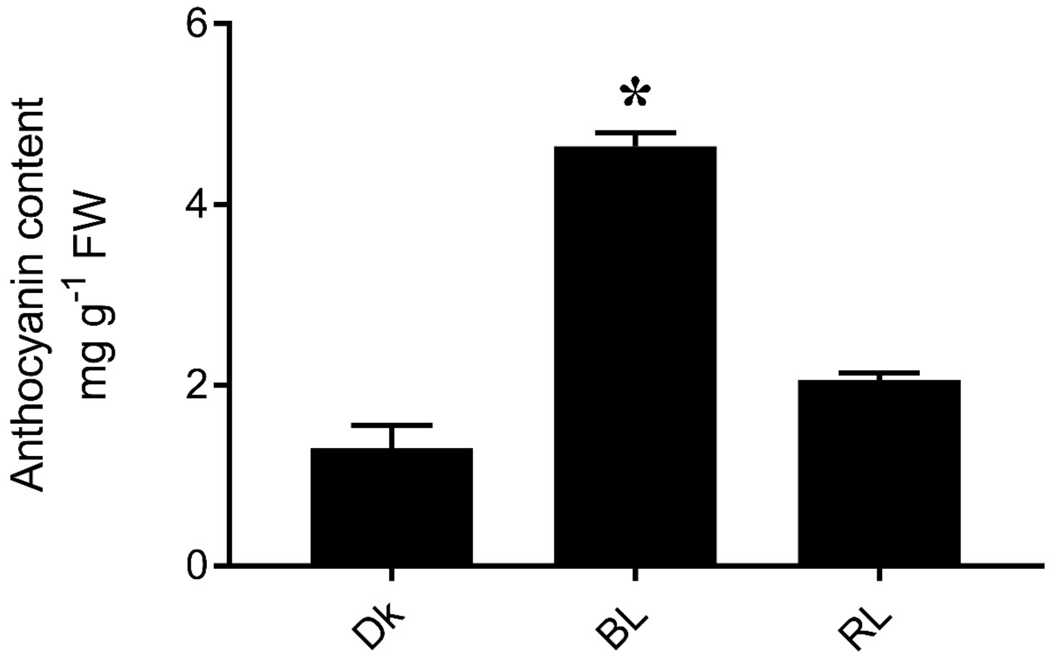

Figure S2

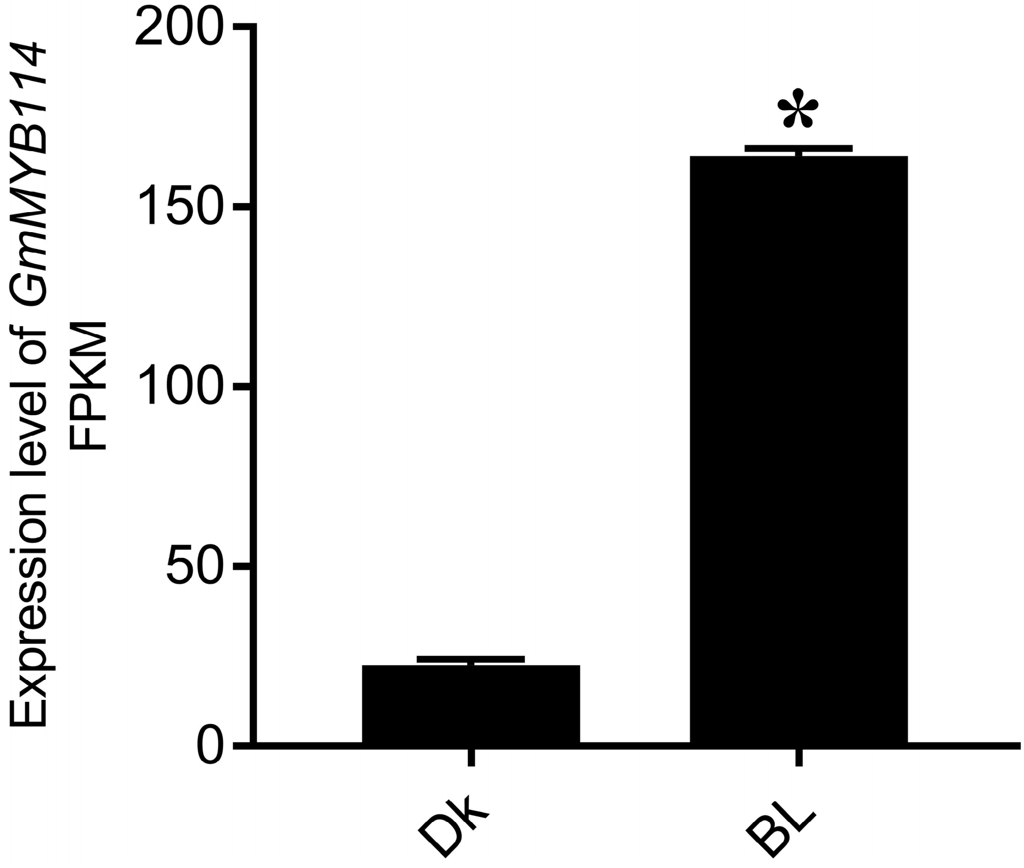

Figure S3

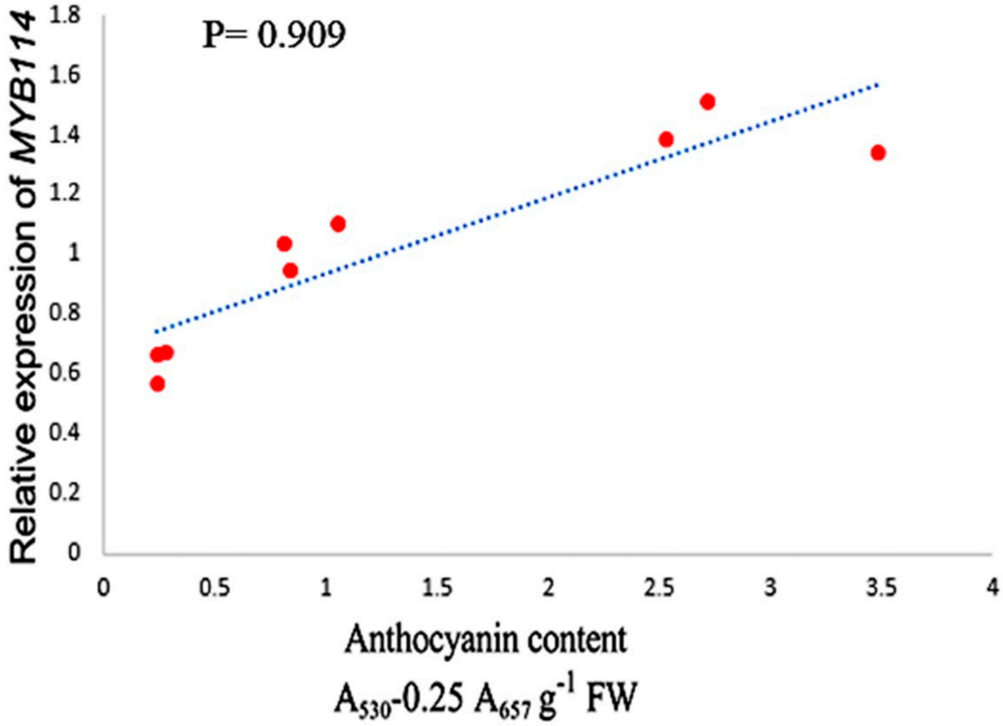

P= 0.909
Relative expression of MYB114
1.8
1.6
1.4
1.2
1
0.8
0.6
0.4
0.2
0
0
0.5
1
1.5
2
2.5
3
3.5
4
Anthocyanin content
A530-0.25 A657 g-1 FW

Figure S4

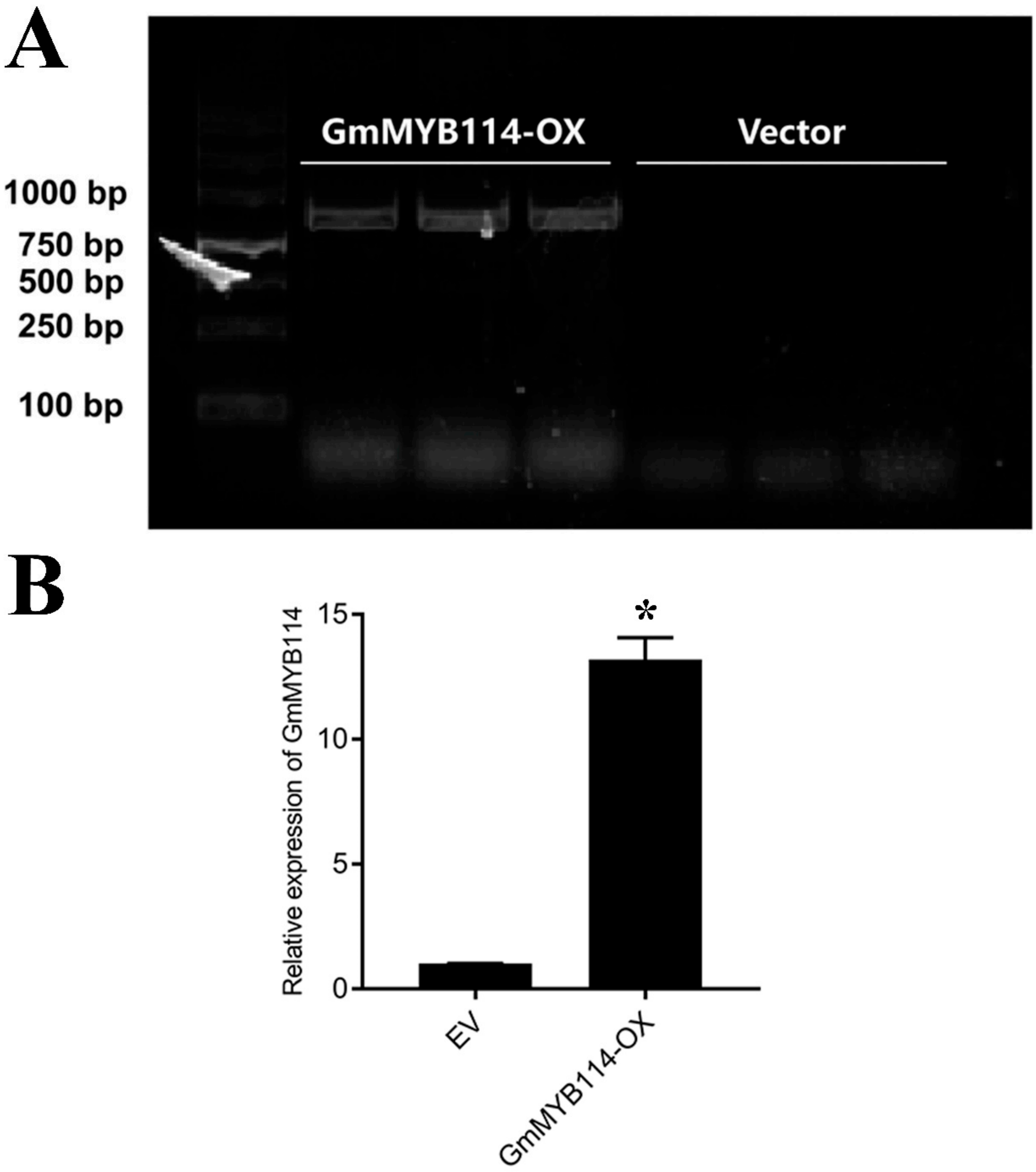
A
GmMYB114-OX
Vector
1000 bp
750 bp
500 bp
250 bp
100 bp
B
Relative expression of GmMYB114
15
10
5
0
*
EV
GmMYB114-OX

Figure S5

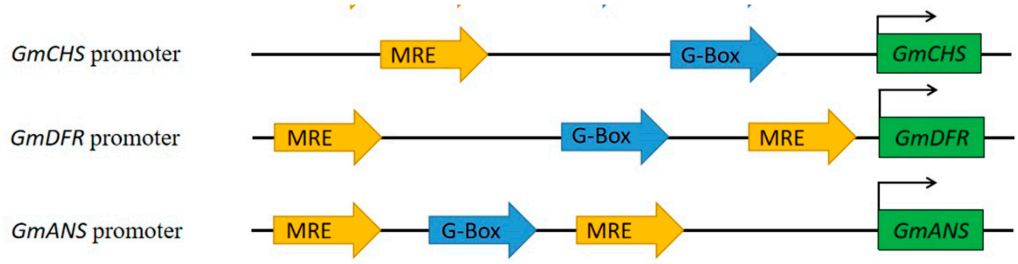

GmCHS promoter
MRE
G-Box
GmCHS
GmDFR promoter
MRE
G-Box
MRE
GmDFR
GmANS promoter
MRE
G-Box
MRE
GmANS
